# Humoral factors in ALS patients during disease progression

**DOI:** 10.1186/s12974-015-0350-4

**Published:** 2015-06-28

**Authors:** Jared Ehrhart, Adam J. Smith, Nicole Kuzmin-Nichols, Theresa A. Zesiewicz, Israt Jahan, R. Douglas Shytle, Seol-Hee Kim, Cyndy D. Sanberg, Tuan H. Vu, Clifton L. Gooch, Paul R. Sanberg, Svitlana Garbuzova-Davis

**Affiliations:** Center of Excellence for Aging & Brain Repair, University of South Florida, Morsani College of Medicine, Tampa, FL USA; Department of Neurosurgery and Brain Repair, University of South Florida, Morsani College of Medicine, 12901 Bruce B. Downs Blvd., Tampa, FL 33612 USA; Department of Molecular Pharmacology and Physiology, University of South Florida, Morsani College of Medicine, Tampa, FL USA; Department of Pathology and Cell Biology, University of South Florida, Morsani College of Medicine, Tampa, FL USA; Department of Psychiatry, University of South Florida, Morsani College of Medicine, Tampa, FL USA; Department of Neurology, University of South Florida, Morsani College of Medicine, Tampa, FL USA; Saneron CCEL Therapeutics, Inc., Tampa, FL USA

**Keywords:** ALS patients, Humoral factors, Cytokines, Nitrite, Glutathione

## Abstract

**Background:**

Amyotrophic lateral sclerosis (ALS) is a neurodegenerative disease affecting upper and lower motor neurons in the CNS and leading to paralysis and death. There are currently no effective treatments for ALS due to the complexity and heterogeneity of factors involved in motor neuron degeneration. A complex of interrelated effectors have been identified in ALS, yet systemic factors indicating and/or reflecting pathological disease developments are uncertain. The purpose of the study was to identify humoral effectors as potential biomarkers during disease progression.

**Methods:**

Thirteen clinically definite ALS patients and seven non-neurological controls enrolled in the study. Peripheral blood samples were obtained from each ALS patient and control at two visits separated by 6 months. The Revised ALS Functional Rating Scale (ALSFRS-R) was used to evaluate overall ALS-patient functional status at each visit. Eleven humoral factors were analyzed in sera. Cytokine levels (GM-CSF, IL-1β, IL-2, IL-4, IL-5, IL-6, IL-8, IL-10, and TNF-α) were determined using the Bio-Rad Bio-Plex® Luminex 200 multiplex assay system. Nitrite, a breakdown product of NO, was quantified using a Griess Reagent System. Glutathione (GSH) concentrations were measured using a Glutathione Fluorometric Assay Kit.

**Results:**

ALS patients had ALSFRS-R scores of 30.5 ± 1.9 on their first visit and 27.3 ± 2.7 on the second visit, indicating slight disease progression. Serum multiplex cytokine panels revealed statistically significant changes in IL-2, IL-5, IL-6, and IL-8 levels in ALS patients depending on disease status at each visit. Nitrite serum levels trended upwards in ALS patients while serum GSH concentrations were drastically decreased in sera from ALS patients versus controls at both visits.

**Conclusions:**

Our results demonstrated a systemic pro-inflammatory state and impaired antioxidant system in ALS patients during disease progression. Increased levels of pro-inflammatory IL-6, IL-8, and nitrite and significantly decreased endogenous antioxidant GSH levels could identify these humoral constituents as systemic biomarkers for ALS. However, systemic changes in IL-2, IL-5, and IL-6 levels determined between visits in ALS patients might indicate adaptive immune system responses dependent on current disease stage. These novel findings, showing dynamic changes in humoral effectors during disease progression, could be important for development of an effective treatment for ALS.

## Background

Amyotrophic lateral sclerosis (ALS) is a neurodegenerative disease that affects motor neurons in the brain and spinal cord leading to paralysis and eventually death, usually within 3 to 5 years of diagnosis. There are two recognized variants of ALS, sporadic ALS (SALS) and familial ALS (FALS). The vast majority (90–95 %) of all ALS cases is sporadic (SALS) while the remaining FALS cases are genetically linked [1, 2]. Within FALS cases, approximately 20 % result from missense mutations in the gene coding for Cu/Zn superoxide dismutase (SOD1) [3, 4] and about 2–5 % have mutations of the TARDBP gene encoding the TAR-DNA-binding protein TDP-43 [5]. Interestingly, mutations in the TARDBP gene have been found in both FALS and SALS [6, 7]. The clinical presentation and underlying pathology of SALS and FALS are similar, and treatment options for ALS patients are limited and mainly supportive. Current drug therapy, such as riluzole (Rilutek®), extends the lifespan of ALS patients by only a few months [8, 9].

Although numerous hypotheses about the etiopathology of ALS have been proposed (reviewed in [10–14]), the complexity and heterogeneity of factors involved in motor neuron degeneration represent a major challenge to developing effective therapies for ALS. A combination of interrelated effectors such as glutamate excitotoxicity, mitochondria dysfunction, glial cell pathology, impaired axonal transport, protein aggregations, and neurotrophic factor deficits leads to progressive motor neuron degeneration and eventual death.

Additionally, oxidative stress [15–18] and neuroinflammation [19–24] have been implicated in both SALS and FALS motor neuron degeneration and are considered important components in ALS pathogenesis. These effectors have been identified in spinal cords, cerebrospinal fluid (CSF), and blood collected from ALS patients. In CSF, dysregulation in expression of anti- and pro-inflammatory cytokines and growth factors, such as interferon-gamma (IFN-γ), tumor necrosis factor-alpha (TNF-α), interleukins (IL)-6 and IL-10, and vascular endothelial factor (VEGF), have been noted [25]. Systemic inflammatory cytokines such as TNF-α, interferon-beta (IFN-β), and various interleukins exacerbating cognitive and motor dysfunction have been identified in various neurodegenerative diseases, including ALS [26–29]. Moreover, several studies demonstrated specific biomarkers in CSF and plasma which might distinguish patients with ALS from other neurological diseases [4, 25] and even predict ALS prognosis [30]. Su et al. [30] showed that particular cytokines found in plasma of ALS patients predict shorter (IL-1β and IL-12) or longer (IL-10) disease duration and suggested that a lesser degree of inflammation might be associated with longer disease duration. However, it has been noted that biomarkers in plasma, mainly indicating ongoing inflammatory processes in ALS patients, might differ from CSF biomarkers [31].

Nitric oxide (NO), a gasotransmitter, has also been shown as a significant contributor in ALS pathogenesis by glutamate-induced neuronal death (reviewed in [32]). NO-related toxicity promotes inflammation through the formation of peroxynitrite that results in disruption of the mitochondrial respiratory chain, production of reactive oxygen species (ROS), and glutamate toxicity [32]. Elevated NO has also been found in patients with other pro-inflammatory conditions such as arthritis [33] and acute pancreatitis [34]. Reductions in glutathione, a component of the endogenous antioxidant system, have been shown in numerous neurodegenerative diseases such as Parkinson’s disease, Alzheimer’s disease, schizophrenia, and ALS, leading to increased concentrations of ROS (reviewed in [35]). Also, significantly decreased glutathione levels were noted in the blood of patients with Friedreich’s ataxia, suggesting that impairment of glutathione homeostasis involved in free radical cytotoxicity contributes to disease pathogenesis [36]. In ALS, however, the role of glutathione is still unclear. The activity of the enzyme glutathione peroxidase was normal [37] or reduced [38] in the spinal cord or brain of ALS patients and also reduced in the blood of SALS patients [39]. It is important to establish specific systemic biomarkers which can easily be monitored in the clinic, serve as a novel diagnostic tool, or verify treatment efficacy for ALS [40]. Particularly, the identification of humoral markers related to oxidative and/or inflammatory status in ALS may have essential implications. To date, only a few human ALS studies (referenced above) have identified specific systemic effectors; however, levels of these effectors have yet to be correlated with disease progression in ALS patients.

In this study, we endeavor to characterize humoral effectors in ALS patients during disease progression. It is essential to establish which inflammatory and oxidative effectors are present, altered, and/or might reflect disease status. Most importantly, identifying major humoral effectors as biomarkers in ALS is key to improved understanding of disease pathogenesis and development of new therapeutic approaches.

## Methods

### Subjects

Thirteen clinically definite ALS patients (12 males and 1 female, mean age 53.9 ± 2.8 years) and seven healthy controls (3 males and 4 females, mean age 57.7 ± 5.7 years) entered our study. Demographic information of all study subjects is provided in Table 1. ALS patients and control subjects visited the University of South Florida (IRB #103861) clinic twice; each visit was separated by 6 months. The diagnoses of ALS were previously established according to the EI Escorial Word Federation of Neurology criteria [41, 42] at various ALS clinics/centers. The Revised ALS Functional Rating Scale (ALSFRS-R), scored from 0 to 48, was used to evaluate overall patient functional status [43]. The ALSFRS-R score was updated for each patient when blood was drawn. Control subjects had no neurological, immunological, or psychiatric comorbidities.

**Table 1:** Demographic information of ALS patients and healthy controls

	ALS patients	Healthy controls
*n*	13	7
Age (years) mean ± SEM	53.85 ± 2.81	57.71 ± 5.73
	Range, 38–68	Range, 37–69
Sex (male/female)	12/1	3/4
ALSFRS-R mean ± SEM first visit	30.46 ± 1.93	48.0 ± 0.0
	Range, 21–41	
ALSFRS-R mean ± SEM second visit	27.25 ± 2.73	N/A
	Range, 21–38	
Disease duration from onset (months) at first visit mean ± SEM	41.77 ± 7.53	N/A
	Range, 11–96	
Disease duration from diagnosis (months) at first visit mean ± SEM	21.00 ± 4.44	N/A
	Range, 5–53	

### Ethics, consent, and permission

This study was approved by the Institutional Review Board at the University of South Florida (IRB #103861). Each participant in the study signed an informed consent form prior to enrollment.

### Collection and processing of blood specimens

Two sets of peripheral blood samples (~90 ml each set) were obtained by venipuncture from each patient and healthy control at -month intervals in accordance with previously described study protocols [44]. Briefly, blood was drawn and collected into a sterile 10-ml tube with a silicone-coated interior (BD Vacutainer, Serum, REF 367820) containing a clot activator. The 10-mL serum tube was placed in a 37 °C incubator for 60 min, allowing the blood to clot, and then stored overnight at 4 °C. The next day, the supernatant was decanted into a 15-mL sterile conical centrifuge tube and centrifuged at 400×*g* for 10 min at 4 °C. After centrifugation, sera transferred to sterile 1.5-mL tubes and stored at −20 °C until assayed. Additionally, a portion of each collected blood sample was sent to the Oklahoma Blood Institute (Oklahoma, OK) for infectious disease (HIV, hepatitis B and C, syphilis, CMV, and HTLV I&II) testing.

### Humoral cytokine detection

A human ultrasensitive cytokine 10-plex panel (Invitrogen; LHC6004) was employed to determine various cytokine concentrations within the sera samples from ALS patients and controls. All cytokine measurements were performed blind by independent investigators to avoid subjective bias. Granulocyte-macrophage colony-stimulating factor (GM-CSF) and cytokine levels (IL-1β, IL-2, IL-4, IL-5, IL-6, IL-8, IL-10, and TNF-α) were determined using the Bio-Rad Bio-Plex® Luminex 200 multiplex assay system. Briefly, a dilution of capture antibody-labeled beads was added to a 96-well filter bottom plate and washed twice with the supplied wash buffer. Washing steps were performed according to the manufacturer’s protocol using vacuum manifold. Samples (1:2 dilution) or standards (supplied in the kit) were then added to their respective wells in duplicate. The plate was then sealed with an adhesive strip and incubated on a plate shaker for 2 h at room temperature (RT). After incubation, the plate was washed twice with the supplied wash buffer and the diluted biotin conjugated detection antibody added. The plate was sealed and allowed to incubate again for 1 h, shaking at RT. Then the wells were washed twice with wash buffer, and diluted streptavidin-RPE reporter reagent (diluted following manufacturer’s instructions) was added to all wells. The plate was incubated again for 30 min while shaking at RT. Finally, the plate was washed three more times with buffer to remove unbound streptavidin-RPE. The analtye/bead complex was then resuspended in 100 μL of wash buffer by shaking for 5 min before analysis. Standard and sample cytokine concentrations were then calculated using the Bio-Rad Bio-Plex® 200 software and results displayed as pg of analyte per mL of sera.

### Serum nitrite detection

Nitrite (NO_2_^−^), a stable and nonvolatile breakdown product of NO, was measured in sera samples using a Griess Reagent System (Promega, Madison, WI) as described previously [33, 45] and per the manufacturer’s instructions. The nitrite measurements were performed blind by independent investigators to avoid subjective bias. Briefly, nitrite standards were prepared in triplicate in fetal bovine serum diluent. The 50 μL of each serum sample were dispensed into a 96-well plate. Sulfanilamide solution (50 μL) was added to each well using a multichannel pipettor, and the plate was incubated for 10 min at RT. Next, 50 μL of *N*-1-naphthyl ethylenediamine dihydrochloride solution was added to each well. Following a 10-min incubation at RT, absorbance was measured using a plate reader (Spectramax Plus 384) set to a wavelength of 530 nm. Nitrite concentrations (μM) were determined by comparing absorbance values to the standard curve.

### Serum glutathione detection

Glutathione (GSH) concentrations were measured using a Glutathione Fluorometric Assay Kit (BioVision, cat. K264-100, Milpitas, CA) according to manufacturer’s instructions. The GSH measurements were performed blind by independent investigators to avoid subjective bias. Briefly, 60 μL of serum was added in 20 μL of 6 N Perchloric acid (PCA). Samples were put on ice for 5 min and centrifuged for 2 min at 13,000×*g* at 4 °C, and 40 μL of supernatant was collected for assay. To precipitate PCA, 20 μL of 6 N potassium hydroxide was added to samples and incubated on ice for 5 min. Samples were then centrifuged for 2 min at 13,000×*g* at 4 °C, and 10 μL of supernatant were transferred to a 96-well plate. To detect GSH, samples were brought up to 90 μL with assay buffer and incubated with 10 μL of o-phthalaldehyde probe for 40 min at RT. Fluorescence readings were measured using a fluorescence plate reader (Spectramax Gemini EM) at 340/420 nm (Ex/Em wavelength). Results are displayed as μg/mL.

### Statistical analysis

Data are presented as means ± S.E.M. Statistical analysis was performed using GraphPad Prism software (GraphPad Software, Inc.). Two-tailed *t*-tests were used, with *p* < 0.05 considered significant.

## Results

### Subject demographics

Blood from 13 ALS patients and 7 healthy controls was collected at two visits, 6 months apart. Patients had been diagnosed with ALS for 21.0 ± 4.44 months (range, 5–53 months) with ALSFRS-R scores of 30.46 ± 1.93 on the first visit. Twelve ALS patients were Caucasian and one was African American. The mean time since the onset of ALS symptoms in this patient population was 41.77 ± 7.53 (range, 11–96) months. After 6 months, ALSFRS-R scores of ALS patients decreased to 27.25 ± 2.73 (Table 1) indicating slight progression of disease. Although there was no significant difference in ALSFRS-R scores of ALS patients (*p* < 0.08) between visits, a substantial decrease in ALSFRS-R scores (6–9 points) was determined in six patients. Scores were unchanged in three ALS patients. Each healthy control scored 48. ALS patients were taking a standard dose of riluzole. One patient refused to take riluzole. Eleven ALS patients had sporadic disease onset at upper, lower, or both limbs. Two patients had familial ALS. No patients had bulbar onset. All study participants tested negative for infectious diseases (HIV, hepatitis B and C, syphilis, CMV, and HTLV I and II).

### Humoral cytokine profile

**Fig. 1:**
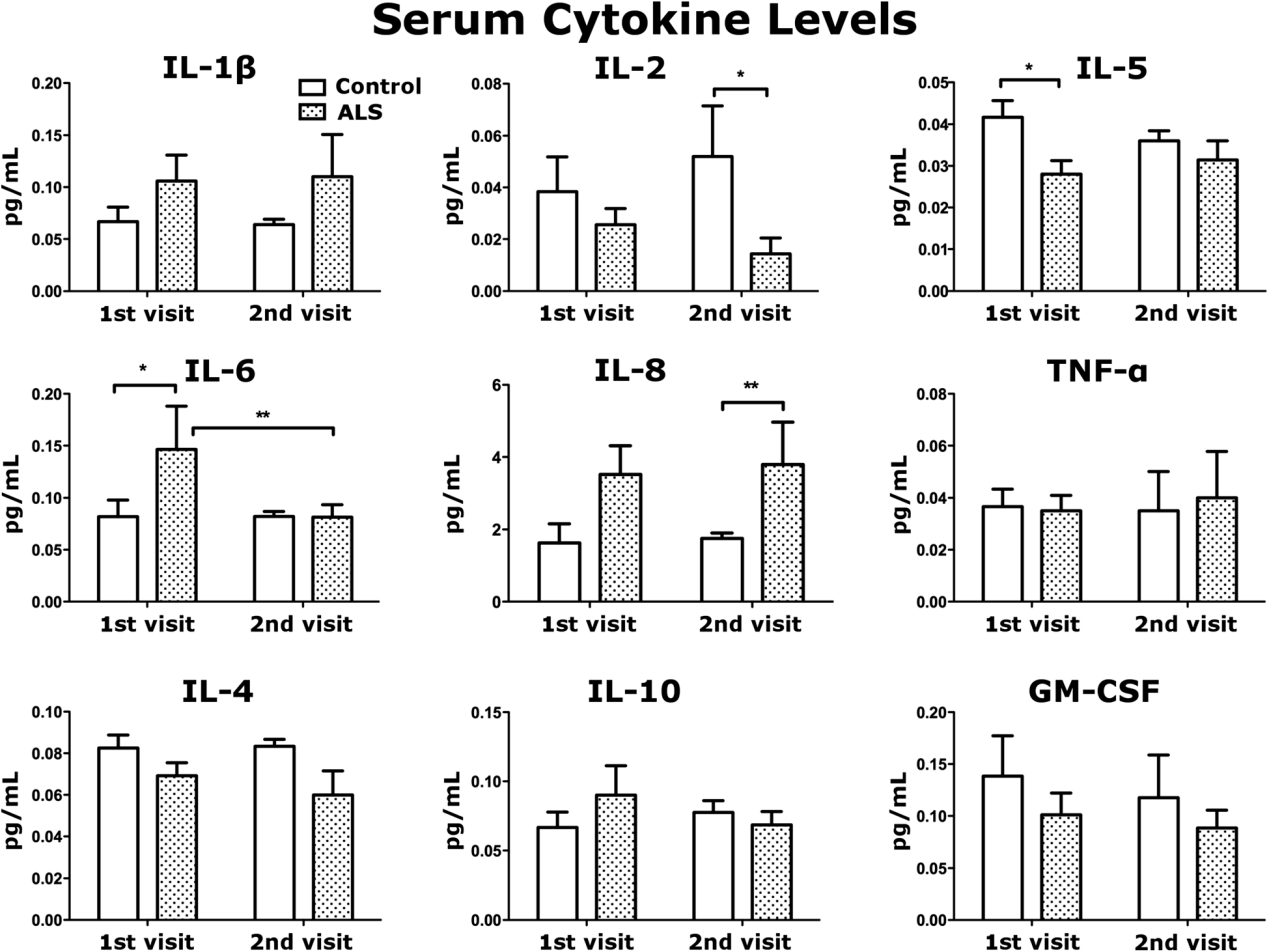
Serum multiplex cytokine panel. Cytokine profile was assayed in sera of ALS patients and controls on both visits using an ultrasensitive human cytokine panel. At first visit, a significant increase in serum IL-6 (*p* < 0.05) and decrease in IL-5 (*p* < 0.05) levels were determined for ALS patients versus controls. At second visit, ALS patients had significantly less serum IL-2 (*p* < 0.05) and IL-6 (*p* < 0.01) although IL-8 increased (*p* < 0.01) compared to controls. There were no significant differences in IL-1β, TNF-α, IL-4, IL-10, and GM-CSF between ALS patients and controls, although elevated IL-1β and decreased IL-4 and GM-CSF were noted in both visits. Results are plotted as mean ± SEM. Statistical significance was determined using two-tailed *t*-tests (* *p* < 0.05, ** *p* < 0.01)

Serum samples from ALS patients and controls were assayed using an ultrasensitive human cytokine 10-plex panel. At the first visit, significant (*p* < 0.05) down-regulation of IL-5 and up-regulation of IL-6 levels were detected in sera of ALS patients compared to controls (Fig. 1). Elevated serum IL-1β and IL-8 levels were determined in ALS patients, although not differing significantly (*p* > 0.05) from control subjects. Also, non-significant decreases (*p* > 0.05) of IL-2 and GM-CSF levels were observed in ALS patients versus controls.

At the second visit (6 months later), serum cytokine profile in ALS patients changed (Fig. 1). Significant (*p* < 0.05) decreases of IL-2 levels showed in sera of ALS patients compared to controls. In contrast to the first visit, IL-5 levels in sera of ALS patients were similar to levels of control subjects. Serum IL-6 levels in ALS patients were also similar to control levels, being significantly (*p* < 0.01) reduced versus initial ALS-patient visits. Significant (*p* < 0.01) increases of IL-8 cytokine levels were found in sera of ALS patients. Similarly to the first visit, non-significantly elevated IL-1β levels were observed in sera of ALS patients after 6 months (*p* > 0.05). Serum TNF-α levels were also similar in ALS patients at both visits compared to controls. Although IL-4 levels were slightly reduced in sera of ALS patients at both visits, these decreases were not statistically significant (*p* > 0.05). Also, there were no significant differences in serum IL-10 levels at either visit between patients and controls (Fig. 1).

### Humoral nitrite levels

**Fig. 2:**
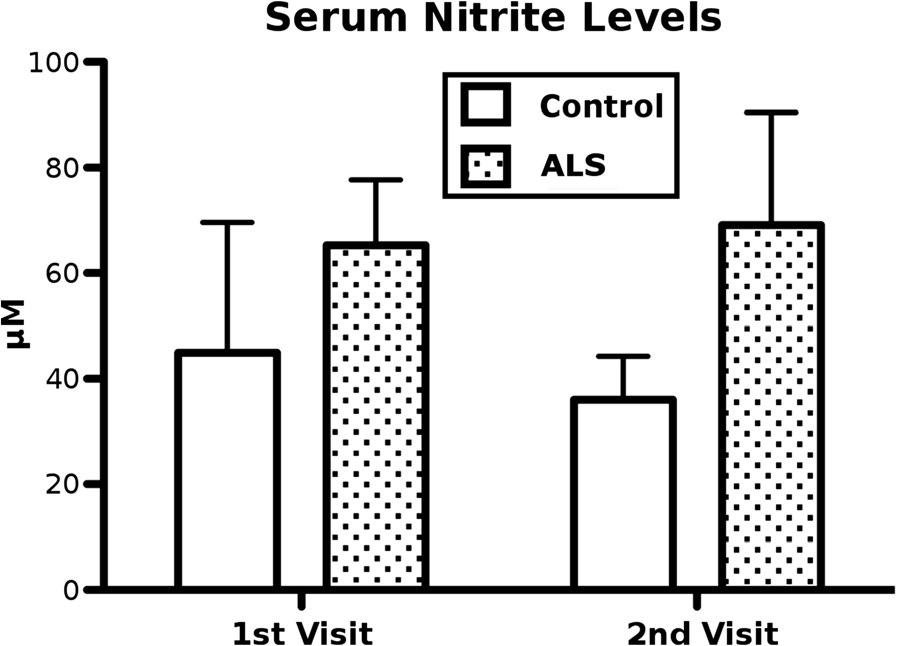
Serum nitrite levels. Nitrite concentrations were assayed in sera of ALS patients and controls at both visits using a Griess Reagent System. Nitrite levels were higher in ALS sera than control sera on both visits, but the differences were not statistically significant (*p* = 0.09). Results are plotted as mean ± SEM. Statistical significance was determined using two-tailed *t*-tests (* *p* < 0.05, ** *p* < 0.01)

Serum samples were assayed to measure concentrations of nitrite, a breakdown product of NO. At both visits, sera from ALS patients contained higher concentrations of nitrite (first visit—65.33 ± 12.38 μM, second visit—69.12 ± 21.29 μM) than the control sera (first visit—44.94 ± 9.32 μM, second visit—36.01 ± 3.70 μM) (Fig. 2). However, concentration was higher in ALS sera with a slight increase at the second visit of ALS patients; differences versus controls were not statistically significant (*p* = 0.09) at either visit.

### Humoral glutathione levels

**Fig. 3:**
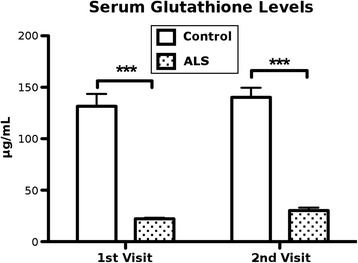
Serum glutathione levels. Glutathione (GSH) concentrations were assayed in sera of ALS patients and controls at both visits using a Glutathione Fluorometric Assay Kit. Dramatically lower GSH levels (*** *p* < 0.001) were determined in sera of ALS patients at both visits compared to controls. Results are plotted as mean ± SEM. Statistical significance was determined using two-tailed *t*-tests (*** *p* < 0.001)

Serum samples were assayed to measure glutathione (GSH) concentrations using a Glutathione Fluorometric Assay. Results showed that sera from ALS patients had drastically lower GSH levels (first visit—22.25 ± 0.99 μg/mL, second visit—30.33 ± 2.68 μg/mL) compared to controls (first visit—131.54 ± 12.05 μg/mL, second visit—140.25 ± 9.31 μg/mL) at both visits (*p* < 0.001) (Fig. 3).

## Discussion

In the present study, humoral factors, as potential biomarkers of ALS, were examined in ALS patients during disease progression. Various pro- and anti-inflammatory cytokines including GM-CSF, nitrite, and glutathione concentrations in sera of ALS patients and age-matched control subjects were evaluated after each of two visits, 6 months apart. The major findings in our study were as follows: 1) significant *increase* of IL-6 in ALS patients at the first visit was not noted at the second visit; 2) *increase* of IL-8 levels was significantly pronounced at the second visit of ALS patients; 3) initial *decrease* of IL-2 was significantly lower in ALS patients at the second visit compared to healthy controls; 4) significant *decrease* of IL-5 levels in ALS patients at the first visit was not present at the second visit; 5) *elevated* nitrite concentrations were determined in both visits of ALS patients; 6) significantly *lower* glutathione concentrations were found in ALS patients at both visits. These results suggest that specific humoral factors might represent inflammatory and oxidative stress responses in ALS with dynamic changes during disease progression.

Comprehensive studies have demonstrated a central role for inflammation in motor neuron degeneration and death in ALS. Large numbers of activated microglia and reactive astrocytes along with infiltration of inflammatory cells have been observed in the brainstem and spinal cord in both ALS patient and animal models [20, 24, 46–53]. These inflammatory effectors are capable of secreting numerous cytokines playing key roles in the inflammatory reaction leading to motor neuron damage. Although sufficient evidence exists of inflammatory reactions in ALS, the role of inflammation in the selective motor neuron death occurring during disease progression in ALS is still unclear and might depend upon disease stage. However, studies have demonstrated a neuroprotective role of inflammation in slowing disease progression by involvement of T-regulatory (Treg)/Th2 immune system activity [54–57]. This activity contributed to anti-inflammatory neuroprotective immune responses that regulated and/or opposed deleterious pro-inflammatory-mediated ALS progression. In both animal models and ALS patients, the endogenous T lymphocytes, specifically Treg cells, increased at an early slowly progressing disease stage, and numbers decreased in conjunction with rapid ALS progression, likely due to the loss of FoxP3, a transcription factor for Treg function [54, 58]. Thus, endogenous Treg lymphocytes actively contribute to neuroprotection in ALS likely via interactions with protective M2 microglia and increased levels of these T cells in ALS patients might have therapeutic value and “slow the rate of disease progression and stabilize patients for longer periods of time” [58]. Though, establishing specific interactions between central and peripheral inflammatory processes in ALS might be critical not only for understanding additional disease mechanisms, but may also have “important implications for therapy” (reviewed in [24]). To support this notion, Zhang et al. [59] found increased expression of HLA-DR (a MHC class II cell surface receptor) on monocytes and macrophages in the blood of SALS patients relating to the rate of disease progression, suggesting a link between systemic macrophage activation and ongoing CNS pathogenic processes.

The elevated cytokines found in sera from ALS patients are predominantly secreted by activated monocytes/macrophages and might be important mediators of the peripheral inflammatory response either promoting a neuroprotection or accelerating disease progression. Elevated IL-6 cytokine levels have been noted in sera from ALS patients [60]. Also, levels of this cytokine were significantly higher in CSF from ALS patients than from patients with other neurological diseases [61]. Our study confirms previous results [60], noting significant increases of IL-6 levels in ALS-patient sera only at the first visit. However, IL-6 was reduced to control levels by the second visit. This interleukin is a bi-functional cytokine, which can act as a pro-inflammatory cytokine and an anti-inflammatory myokine. IL-6 is secreted by T lymphocytes and macrophages during infection, stimulating immune response [62–64] and serving as a pro-inflammatory mediator. The anti-inflammatory role of IL-6 has been mainly associated with muscle contractions [65, 66] but likely this is not the case in ALS. Possibly, this cytokine initially acts as a pro-inflammatory protein in ALS-patient sera and, upon disease progression, becomes an unreliable marker of peripheral inflammation. However, since IL-6 can cross the blood-brain barrier [67], the significant IL-6 sera levels determined at the first visit of ALS patients might have been abrogated at the second visit due to infiltration of this protein per se in addition to extravasation of inflammatory cells into the CNS parenchyma. Also, it has been shown that IL-6 increases might be related to the hypoxia experienced by some ALS patients and may not indicate inflammatory status as previously suspected [68]. Measurements of blood oxygen levels in ALS patients might be informative in this respect and merit further investigation.

In accord with significantly elevated IL-6 levels in sera from ALS patients at the initial visit, IL-8 levels also increased and substantially escalated at the second visit. The IL-8 is a member of the CXC chemokine family and is produced by macrophages and other cell types, including endothelial cells [69]. This chemokine is primarily associated with inflammation and is known as a pro-inflammatory mediator [70] and/or mediator of immune reaction in the innate immune system response [71]. Although IL-8 predominantly has chemoattractive activity for neutrophils, this chemokine is also a potent angiogenic factor [72]. In a study by Kuhle et al. [73], IL-8 and monocyte chemoattractant protein-1 (MCP-1) levels were shown to be significantly higher in CSF from ALS patients versus control (non-inflammatory neurological disease) patients. However, serum levels of these chemokines did not significantly differ between ALS patients and controls. The authors concluded that increased IL-8 levels in CSF suggested “stimulation of pro-inflammatory cytokine cascade after microglia activation” and that high CSF-MCP-1 levels might be associated with rapid disease progression and short survival time. Nevertheless, use of these chemokines as prognostic factors for ALS was not investigated in this study, as the chemokines were analyzed in CSF and sera from ALS patients only once at early stage of disease (281 days from first symptoms to diagnosis). In our study, increased IL-8 levels in sera correlated with disease stage, suggesting that this durable pro-inflammatory mediator might be a useful marker of inflammation in ALS progression.

Another finding of our study was that IL-2 and IL-5 levels were lower in ALS-patient blood during both visits with significant decreases in the first (IL-5) and second (IL-2) visits. IL-2 is an immunoregulatory lymphokine, which is produced by T cells [74]. This cytokine regulates lymphocyte activity in the immune system, mainly in response to infection, and promotes the differentiation of immature T cells into both pro-inflammatory T cell subsets in the Th1/Th17 pathways, resulting in acceleration of disease progression, and anti-inflammatory regulatory T cells which maintain cellular immunologic memory to protect the host from reinfection by the same pathogen [75]. IL-5 is mainly produced by T helper (Th2) and mast cells and stimulates B cell growth and activation, increasing antibody production [76]. It is also involved in eosinophil activation [77, 78] in association with several allergic diseases [79–81]. In ALS patients, no significant differences in serum IL-2 levels versus control subjects were reported [82, 83] suggesting that this cytokine might have a limited input in the immune system response, at least in the early stage of disease (14.3 ± 9.34 months disease duration in [83]). In contrast, the significantly decreased IL-2 in sera of ALS patients at a more pronounced disease stage (42 months disease duration) demonstrated in our study strengthens potential down-regulation of this cytokine on differentiation and/or maintenance of all T cell subsets, including regulatory T cell populations, such as T helper cells, that might lead to a Th1/Th2 imbalance toward the development of an autoimmune disorder [84]. A reduction in IL-2 cytokine expression can affect not only the proliferation of pro-inflammatory Th1/Th17 cell subsets promoting ALS progression, but can also increase Treg/Th2 subsets that provide anti-inflammatory stimuli resulting in protection during an inflammatory event. Also, since a significant reduction of regulatory T cells (CD4 + CD25+) and monocytes (CD14+) in blood of sporadic ALS patients with less severe disease was noted [85], suggesting early recruitment of these cells to the CNS areas of neurodegeneration, it is possible that decreased serum IL-2 levels in our ALS patients during disease progression reflects limited guidance of antigen-driven T cell development. The role of IL-5 in systemic circulation of ALS patients, however, is unclear. Various cytokines are known to modulate the balance between humoral and cellular immune responses depending on eminence of antigen activation of B and T lymphocytes into antibody-producing plasma and T effector cells. For instance, abnormally high immunoglobulin G (IgG) concentrations and IgM/IgA levels within normal ranges were indicated in sera from ALS patients with early or moderate disease stages [44, 86, 87]. However, some studies demonstrated that serum IgG, IgM, or IgA in ALS patients did not differ from control levels [59, 88] or found even lower IgG concentrations in patients with severe disease stage [59]. These conflicting reports on serum antibody concentrations in ALS might identify immunological profiles varying with predominant humoral or cellular immune response. However, our previous data [44] showing highly elevated serum circulating immune complexes and IgG levels in sporadic ALS patients during disease progression suggest that a humoral immune response might initiate adaptive immunity in ALS. It is possible that the significant decrease of serum IL-5 in ALS patients at the first visit shown in our current study indicated suppression of B cell activation and/or antibody production, likely due to the existing large amount of IgG in sera.

Furthermore, our study results showed that levels of pro-inflammatory cytokines IL-1β and TNF-α in sera from ALS patients did not significantly differ from controls, although they were elevated during disease progression. Also, no significant differences were noted in anti-inflammatory IL-4 and IL-10 cytokine concentrations between ALS patients and control subjects. These results conflict with previous reports demonstrating increased TNF-α and IL-1β levels in the blood of ALS patients [89–91]. However, our findings showing mostly analogous serum IL-1β and TNF-α levels between ALS patients and controls supported the recent study of Furukawa T et al. [83], demonstrating similar outcomes. Furthermore, the infiltration of IL-1β- and TNF-α-positive macrophages detected in the spinal cord of sporadic ALS patients but not in controls [28] might possibly explain the low levels of these cytokines observed in the sera of our ALS patients.

In addition to cytokine/chemokine profiling in systemic circulation of ALS patients during disease progression, nitrite and glutathione (GSH) were evaluated as potential biomarkers for this disease. Elevated nitrite and significantly reduced GSH levels determined in sera of ALS patients versus controls on both visits in our study might indicate ongoing oxidative stress, likely due to prolonged pro- and anti-oxidative imbalances [92]. Increased NO and reduced oxidized glutathione levels detected in CSF from sporadic ALS patients suggest that NO production is activated in ALS and leads to reductions in superoxide radicals that normally oxidize glutathione [93]. Also, impairment in activity of glutathione peroxidase, the predominant antioxidant enzyme, was noted in plasma from sporadic ALS patients, and levels remained low during disease course [94]. Although numerous studies showed that oxidative stress plays a substantial role in motor neuron degeneration in ALS [15–18, 95], some evidence points to oxidative stress as “not just an event in the CNS but rather a systemic process” leading to the suggestion that “ALS is possibly a systemic disease” [96]. Biomarkers of oxidative or nitrosative stress were determined not only in the CNS tissue but also in plasma, urine, and CSF of ALS patients [97–99]. Moreover, increased oxidative stress and immune activation correlating with disease progression were demonstrated in serum and CSF of sporadic ALS patients [98]. Of note, both GSH and NO levels can fluctuate due to a variety of dietary and environmental factors [100, 101] and are thus likely non-specific disease indicators. Glutathione production itself is directly dependent on the presence of the α-amino acid cysteine. Essentially, if adequate concentrations of cysteine are not produced in the body or acquired through diet, then the major constituent of the endogenous antioxidant system cannot be synthesized, leaving unprocessed reactive oxygen species in the body and resulting in oxidative damage to cells and tissues. The GSH and NO levels observed in the systemic compartment in ALS patients during disease progression lends further credence to the idea that oxidative stress is an important hallmark of the pathological changes in ALS.

Numerous studies showed that immune reactivity, inflammatory processes, and oxidative stress are actively involved in ALS pathogenesis (reviewed in [22, 23, 96, 102, 103]). However, it is uncertain which factor(s) trigger immune, inflammatory systemic response, and/or pro-oxidative state in this disease. Also, the role of the immune system as a primary mechanism of motor neuron degeneration in ALS and/or a secondary response to inflammation is still unclear. It is known that an innate immune response is initiated by microglia activation in ALS and an adaptive immune response might be associated with inflammation in the CNS. Thus, the systemic compartment could largely reflect the complexity of the ALS immune/inflammatory response, including oxidative stress, depending on disease stage. In support of this notion, Mantovani et al. [85] provided evidence of immunological alterations in the blood of ALS patients associated with ongoing neuroinflammatory processes. Neuroinflammation in ALS is believed to be a prominent pathological event in areas of motor neuron degeneration. Both innate and adaptive immune responses might highly influence disease progression by shifting from beneficial (neuroprotective) to deleterious (neurotoxic) immune responses. However, McCombe and Henderson [23] noted that “It is possible that individual variability in immune responsiveness means that individual patients have different immune responses in ALS.” A clear understanding of “the dynamic changes that occur within the immune system over the course of disease” [104] is essential for developing an effective treatment for ALS.

In conclusion, results of the present study indicate a clear shift toward a systemic pro-inflammatory state and impaired antioxidant system in ALS patients during disease progression. This is evidenced by increased pro-inflammatory humoral factors such as IL-6, IL-8, and NO and decreased antioxidant glutathione levels. These constituents could serve as systemic biomarkers of ALS. However, systemic changes in some interleukin (IL-2, IL-5, and IL-6) levels determined in the same cohort of ALS patients might indicate specific adaptive immune system responses depending on the current disease stage. Although the concurrence of inflammation, oxidative stress, and immune system alterations in the systemic compartment of ALS patients needs elucidation, the present study supports the notion that monitoring dynamic changes of humoral effectors during disease progression may further our understanding of pathological alterations in ALS. Also, our novel findings should be considered for endeavors to develop an effective treatment for ALS.

## Abbreviations

ALS: amyotrophic lateral sclerosis
ALSFRS-R: Revised ALS Functional Rating Scale
CNS: central nervous system
CSF: cerebrospinal fluid
FALS: familial amyotrophic lateral sclerosis
GM-CSF: granulocyte-macrophage colony-stimulating factor
GSH: glutathione
IFN: interferon
Ig: immunoglobulin
IL: interleukin
MCP-1: monocyte chemoattractant protein-1
NO: nitric oxide
PCA: perchloric acid
ROS: reactive oxygen species
RT: room temperature
SALS: sporadic amyotrophic lateral sclerosis
SOD1: Cu/Zn superoxide dismutase
TNF-α: tumor necrosis factor-alpha
VEGF: vascular endothelial growth factor
